# Ocean currents help explain population genetic structure

**DOI:** 10.1098/rspb.2009.2214

**Published:** 2010-02-04

**Authors:** Crow White, Kimberly A. Selkoe, James Watson, David A. Siegel, Danielle C. Zacherl, Robert J. Toonen

**Affiliations:** 1 Marine Science Institute, University of California, Santa Barbara, CA 93106, USA; 2 Institute for Computational Earth System Science and Department of Geography, University of California, Santa Barbara, CA 93106, USA; 3 Hawaii Institute of Marine Biology, University of Hawaii, Kaneohe, HI 96744, USA; 4 Department of Biological Science, California State University, Fullerton, CA 92834, USA

**Keywords:** seascape genetics, dispersal, pelagic larvae, isolation by distance, derived oceanographic distance

## Abstract

Management and conservation can be greatly informed by considering explicitly how environmental factors influence population genetic structure. Using simulated larval dispersal estimates based on ocean current observations, we demonstrate how explicit consideration of frequency of exchange of larvae among sites via ocean advection can fundamentally change the interpretation of empirical population genetic structuring as compared with conventional spatial genetic analyses. Both frequency of larval exchange and empirical genetic difference were uncorrelated with Euclidean distance between sites. When transformed into relative oceanographic distances and integrated into a genetic isolation-by-distance framework, however, the frequency of larval exchange explained nearly 50 per cent of the variance in empirical genetic differences among sites over scales of tens of kilometres. Explanatory power was strongest when we considered effects of multiple generations of larval dispersal via intermediary locations on the long-term probability of exchange between sites. Our results uncover meaningful spatial patterning to population genetic structuring that corresponds with ocean circulation. This study advances our ability to interpret population structure from complex genetic data characteristic of high gene flow species, validates recent advances in oceanographic approaches for assessing larval dispersal and represents a novel approach to characterize population connectivity at small spatial scales germane to conservation and fisheries management.

## 1. Introduction

Management and conservation efforts can benefit from considering explicitly how environmental factors influence population connectivity and patterns of population genetic structuring. Complex genetic patterns common to marine systems often complicate conclusions regarding connectivity using conventional spatial genetic analyses (Bradbury & Bentzen 2007). A widely used population genetic model of heterogeneous migration, the stepping-stone model (Kimura 1953; Kimura & Weiss 1964), assumes that dispersal probability declines with distance from the source, reducing the mixing of migrants and creating increased genetic dissimilarity with distance between populations (Wright 1943). A spatially explicit isolation-by-distance analysis assesses the fit of empirical population genetic data with the stepping-stone model by testing for a positive linear relationship between geographical distance and genetic difference (e.g. estimated with population pairwise *F*_ST_) between sampled sites. In many cases, however, no linear fit is found despite high variance in pairwise genetic difference values indicating diversity in population connectivity (Bradbury & Bentzen 2007). A lack of fit may be due to high dispersal leading to low genetic differentiation among population pairs, i.e. panmixia, a recent disturbance to drift–migration equilibrium such as a recolonization event, or because pairwise genetic difference values are driven by a force other than Euclidean distance (Castric & Bernatchez 2003; Manel *et al*. 2003).

In the marine environment, currents can be circuitous and oceanographic features like eddies and fronts can prevent mixing and diffusion of pelagic larvae, decoupling pelagic larval dispersal from Euclidean distance (Weersing & Toonen 2009). Two adjacent sites may rarely exchange migrants if located on different sides of an oceanographic front (Gilg & Hilbish 2003), and two distant sites may be well connected by a strong current between them (Mitarai *et al*. 2009). A model of these oceanographic forces may enhance the interpretation of spatial population genetic patterns otherwise unresolved in relation to the geographical distribution of sites. On coarse spatial scales, incorporating oceanographic information into genetic analysis has proved fruitful for estimating connectivity (Gilg & Hilbish 2003; Baums *et al*. 2006; Galindo *et al*. 2006; Kenchington *et al*. 2006; Dupont *et al*. 2007; Fontaine *et al*. 2007; Schultz *et al*. 2008; Knutsen *et al*. 2009; Yasuda *et al*. 2009). The practice has been coined ‘seascape genetics’, and it borrows techniques from landscape genetics designed to test for environmental drivers of spatial genetic structure (Hansen & Hemmer-Hansen 2007; Storfer *et al*. 2007). Terrestrial landscape genetics has proved to have sufficient resolution to detect structure at fine spatial scales even in cases of high gene flow (Clark *et al*. 2008). In contrast, seascape genetics has to date only resolved structure associated with prominent physical barriers (e.g. a narrow strait, deep channel or prominent headland)—and only in species with relatively low gene flow (e.g. global *F*_ST_ ≥ 0.1).

This study represents an important advancement in the evaluation of spatial marine population genetic data (i.e. seascape genetics) by applying a new approach for incorporating ocean circulation observations directly into the isolation-by-distance analysis. As Rousset (1997) specifies, the isolation-by-distance analysis ‘does not require the definition of subpopulations on a lattice (i.e. consideration of geographic distribution *per se*), but only the knowledge of the (relative) distance between samples’. The challenge then is to use a model of oceanographic forces to map frequencies of dispersal between genetic sampling sites, and translate them into relative distances that can be tested for a linear fit with pairwise genetic difference. We estimated frequencies of larval exchange among populations of a marine species based on simulations of dispersal trajectories of the species' larval stage in a data-assimilated oceanographic circulation model produced from ocean temperature, salinity, current and wind observations in the study area. We focused our analysis on *Kelletia kelletii*, a subtidal whelk in US and Mexico Pacific waters that is a significant predator in kelp forest ecosystems (Halpern *et al*. 2006), a possible indicator species for the onset of El Niño conditions (Zacherl *et al*. 2003) and the focus of both a rapidly increasing fishery (Aseltine-Neilson *et al*. 2006) and a microchemistry study estimating its larval dispersal patterns (Zacherl 2005). Recognizing that population genetic patterns can represent dispersal processes integrated over many generations, we explicitly accounted for connections between pairwise locations in the oceanographic model that were established over multiple generations by multiple dispersal events involving intermediary site(s). The resulting probabilities of dispersal were translated into relative distances and used to interpret the empirical pattern of pairwise genetic differences of *K. kelletii* using the isolation-by-distance framework. Our approach enabled the construction of genetic isolation by ‘derived oceanographic distance’ (DOD) plots that were far more effective at organizing the pattern of population genetic structure than conventional isolation-by-distance methods based on Euclidian distance.

## 2. Material and methods

We evaluated genetic polymorphism of *K. kelletii* at nine microsatellite loci, K13, Kk2b, Kk7a, Kk28a, Kk33a, Kk34a, Kk41a, Kk48b and Kk52a, described and amplified with laboratory methods published previously (White & Toonen 2008), across 10 geo-referenced sampling sites in the Santa Barbara Channel, California, USA (table 1). All genetic material used in our analysis was from adult, reproductively mature whelks 60–150 mm in shell length (5–20+ years old; C. White & D. C. Zacherl 2009, unpublished data). Thus, sampling covers genetic patterns represented by a range of cohorts. Samples were collected using SCUBA (15–30 m depth) during the summers of 2004 and 2005. Tested previously for one of the sites (Yellowbanks; White & Toonen 2008), all loci passed null allele frequency, linkage disequilibrium, selective neutrality and Mendelian inheritance quality control screening (as outlined by Selkoe & Toonen 2006). We repeated null allele frequency, linkage disequilibrium and selective neutrality tests for the nine loci across all 10 of the sampling sites, using population genetics programs Freena, FStat and PyPop, respectively (Goudet 1995; Chapuis & Estoup 2007; Lancaster *et al*. 2007).

**Table 1.** Description, abbreviation code, location, adult sample size used in genetic analyses (*N*), Nei's unbiased gene diversity (Hz), mean number of alleles per locus (MNA) and pairwise connectivity statistics for sampling sites. In connectivity matrix, super-diagonal cells contain multi-generation-derived oceanographic (top cell) and Euclidean (bottom) distances, rounded to the nearest integer, and subdiagonal cells contain pairwise *F*_ST_ (top cell, *p < 0.05) and *D*_est_ (bottom) values, between each pair of sampling sites. Pairwise comparisons among island sites and among mainland sites are highlighted in grey shades. See electronic supplementary material, appendix B for variances in derived oceanographic distances calculated across the 7 simulation years available in the oceanographic model.

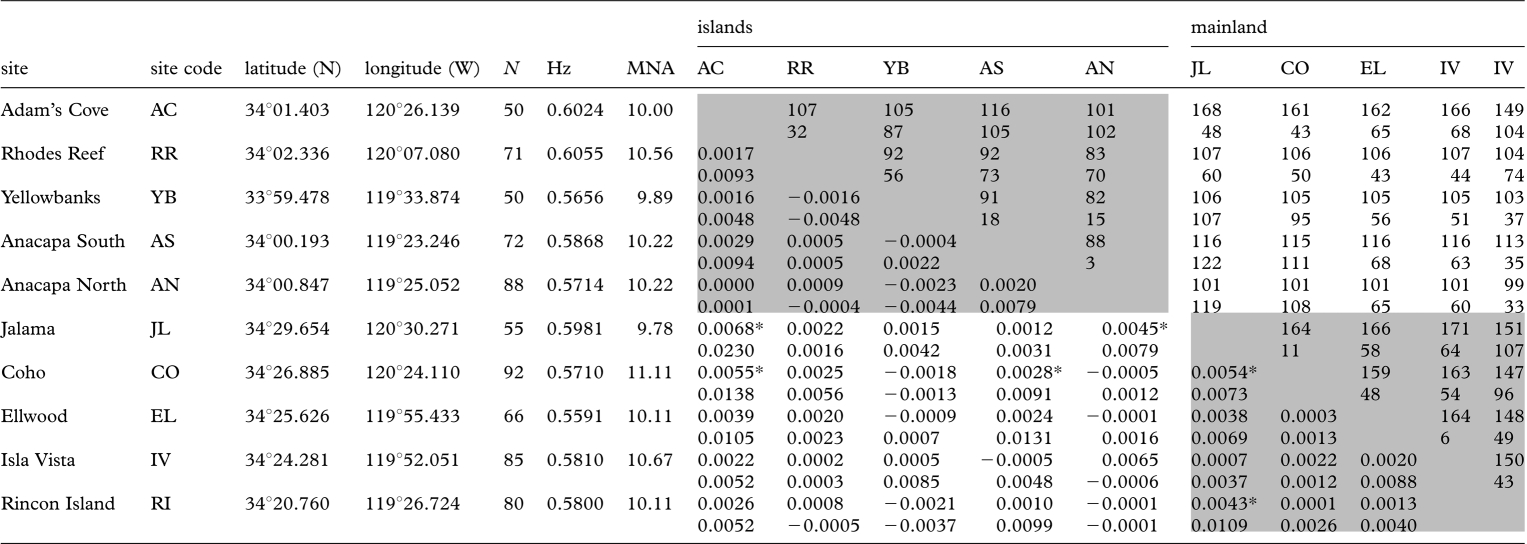

To assess population genetic structure, we estimated global and pairwise genetic differences among sampling sites. We determined population structure using Wright's hierarchical *F*-statistics calculated from the programs FStat and Genetix (Goudet 1995; Bekhir *et al*. 2009). As a fixation index, *F*_ST_ does not accurately measure the magnitude of genetic differentiation among populations when heterozygosity is high and/or variable among sampling locations (Hedrick 2005; Jost 2008). Thus, we compared the estimates of *F*_ST_ with estimates of actual genetic differentiation, *D*_est_, using the program Smogd (Jost 2008; Crawford in press). We also inferred spatial clustering of the geo-referenced, multi-locus individuals using a Bayesian framework applied in program Geneland (Guillot *et al*. 2005); detailed methods are described in electronic supplementary material, appendix A.

Simulations of larval trajectories for the Santa Barbara Channel, California, and the surrounding southern California area were used to estimate probability of larval dispersal and ultimately DOD among genetic sampling sites. Data-assimilated models of ocean currents for the study region were produced by combining available observations of ocean temperature, salinity, currents and winds in a numerical model to provide a dynamic interpolation of the near-surface ocean flow field (see Oey *et al*. 2004 for a full description of the model). The resulting circulation patterns for this region are available on a 5 km spatial horizontal resolution for the period 1993 to 1999. We seeded the flow fields with virtual larvae at 448 5 × 5 km coastal grid locations (electronic supplementary material, figure S1 and appendix A). Twenty-five surface water parcel-following virtual larvae were released daily during *K. kelletii's* seasonal spawning period of larvae in southern California (15 June–15 August, based on field observations; C. White & D. C. Zacherl 2003, unpublished data; thus 448 grids × 25 larvae d^−1^ × 60 days = 672 000 larvae yr^−1^ in total). Particles were advected by simulated currents (e.g. as done by Siegel *et al*. (2008); electronic supplementary material, figure S1*b*) and were allowed to settle during *K. kelletii's* natural age-at-settlement competency window (pelagic larval duration of 40–60 days after release, based on laboratory observations; D. C. Zacherl 2009, unpublished data). Variability in the resulting connectivity matrices reached an asymptote at 25 particles per grid unit per day, prompting our use of this sample size here. To estimate potential larval connectivity, we assumed constant larval production for all release locations and no larval mortality (see §4).

**Figure 1.**
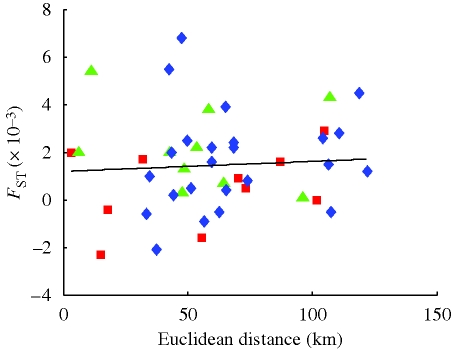
Genetic differentiation in relation to Euclidean distance between sampling sites. See table 2 for regression statistics. Red squares, islands; green triangles, mainland; blue diamonds, cross channel.

Larval dispersal was simulated for 7 years (1993–1999). The observational period included the 1997/1998 El Niño event and thus represented both ‘normal’ and ‘exceptional’ oceanographic conditions. For each year, we recorded the proportion of larvae released from each grid cell that dispersed to a given grid cell. This procedure resulted in a 448 × 448 source–destination (row–column) matrix, **C**_***ji***_, for each simulation year. The value within each matrix indicates the frequency with which the larvae released from near-shore grid cell *j* dispersed with ocean currents to grid cell *i*, given the spawning season and larval settlement competency window.

Estimation of DOD for each pair of genetic sampling locations was done after subjecting the source–destination matrices to five transformations. First, we calculated a single, all-years 448 × 448 matrix as the element-by-element mean of the frequencies in the annual matrices. Second, we standardized the values in the all-years matrix so that for each source, destination probabilities sum to one (i.e. all columns within each row sum to one). This procedure generated a forward transition matrix (Bodmer & Cavalli-Sforza 1968), which now indicates the expected probability of dispersal of *K. kelletii* larvae to grid cell *i* from grid cell *j*.

In the third transformation, we incorporated the effects of multi-generational gene flow on the long-term probability of dispersal between locations. Gene flow data from neutral markers represents a long-term average of dispersal integrated over 10^2^–10^4^ generations, dependent on the demographics, marker mutation rate and other factors (Whitlock & McCauley 1999). In contrast, the current format of the forward transition matrix represents only the average direct, single-generation dispersal events between pairwise locations; it does not capture the effect of multi-generational dispersal processes connecting two sites via other intermediary site(s). We incorporated effects of multi-generational processes into our estimates of dispersal probabilities between sites using a homogeneous Markov chain of matrix multiplication (Bodmer & Cavalli-Sforza 1968; Brémaund 1999). The Markov chain is a convenient theoretical tool used in stepping-stone models that has provided rather satisfactory theoretical explanations to observed long-term patterns of genetic structure of populations (Grinstead & Snell 1998). The Markov chain characterizes transition probabilities between states after a series of independent transition events (Sorensen & Gianola 2002). We used the Markov chain to calculate the probability of dispersal of larvae between grid cells after *n* number of dispersal generations. This multi-generational probability of dispersal between two grid cells is calculated in relation to probabilities of dispersal associated with one to *n* number of single-generation dispersal events involving zero to (*n* − 1) intermediary sites. Thus, the Markov chain captures effects of both single- and multiple-generation dispersal processes on the long-term probability of dispersal among locations. Let **M** represent the original forward transition matrix that was used to set the initial condition. When *n* is sufficiently large, **M**^*n*^ = **M**_**ss**_, the Markov chain output of steady-state (long-term) dispersal probabilities between pairwise locations that is independent of the initial state of the population. We iterated the Markov chain until it reached numerically computed convergence, which took about 10^3^ iterations.

Pairwise *F*_ST_ and *D*_est_ values represent genetic differences between subpopulations without indicating directionality of gene flow. In contrast, both **M** and **M**_**ss**_ contain two values for each pair of locations, each representing a uni-directional probability of dispersal. To correspond with our empirical estimates of population genetic differences between each pair of sampling locations, in the fourth transformation step we averaged uni-directional dispersal probabilities to estimate mean probability of dispersal between pairwise locations. We performed this transformation step for both **M** and **M**_**ss**_ in order to measure the effect of consideration of multi-generational dispersal events (transformation step 3) on our ability to explain the observed pattern of population genetic structure.

The isolation-by-distance analysis tests for a positive linear relationship between genetic differentiation and distance, requiring conversion of the matrix of mean dispersal probability into a distance matrix for the final step of the transformation process. Because each row in the matrix sums to one, the collection of dispersal probabilities for each source can be considered a probability density function (PDF). This type of PDF is termed a ‘larval dispersal kernel’ and provides a mathematical framework for transforming dispersal probabilities into relative dispersal distances (Siegel *et al*. 2003). A dispersal kernel can be generated de novo by simulating larval dispersal along a linear coastline and calculating the probability of dispersal for all pairwise distances between source and destination sites. Siegel *et al*. (2003) derived a dispersal kernel for sites along a linear coastline by simulating larval dispersal in a two-dimensional flow field based on surface drifter and moored current meter observations, and determined how the kernel varies with ocean flow statistics and the duration of the pelagic stage of larvae. Their kernel function enabled us to transform the dispersal probabilities produced here into alongshore distances. This technique linearized the data for use in the isolation-by-distance framework. We term this relative dispersal distance DOD to highlight the ocean flow field as the underlying mechanism connecting dispersing larvae between spawning and settlement locations.

The dispersal kernel of Siegel *et al*. (2003) is Gaussian (normal), with a standard deviation or spread, *σ*_d_, of larval dispersal owing to the fluctuating components of flow, and a downstream offset of the mode of the PDF owing to the mean flow. The spread of the larval dispersal function is relevant here and is calculated knowing the pelagic larval duration, *T*_PLD_, and the root mean square of the fluctuating current velocity, *σ*_*u*_, in the flow field (*σ*_d_, = 2.238*σ*_*u*_*T*^1/2^_PLD_; Siegel *et al*. 2003). The parameter *T*_PLD_ was set to 50 days to match *K. kelletii*'s mean pelagic larval duration. The parameter *σ*_*u*_ was set to 3.4278 km d^−1^, the average among the grid cells in and around the Santa Barbara Channel of the root mean square of the fluctuating current velocity produced in Oey *et al*.'s (2004) circulation model for 15 June–15 August 1993–1999. We based *σ*_*u*_ on a regional average because in the simulations larvae were dispersed by ocean currents throughout the Santa Barbara Channel and the surrounding southern California region (electronic supplementary material, figure S1*b*; as shown by Mitarai *et al*. (2009) as well). The alongshore advection of larvae, the offset of the mode of the dispersal PDF, was set to zero because it was accounted for in the larval dispersal simulations. For the transformation, the probability of dispersal in the PDF was set to the mean probability of dispersal between a pair of grid cells, with the corresponding value in the PDF of mean dispersal distance representing DOD between the two grid cells to dispersing *K. kelletii* larvae. DODs were calculated for mean probabilities of dispersal between the 45 pairs of locations represented by grid cells selected from the flow field that were closest in their centroid position to the 10 geo-referenced genetic sampling sites (electronic supplementary material, figure S1*a*). No two geo-referenced sites were closest to the same centroid; however, if this had been the case, we would have calculated DOD in relation to the mean probability of dispersal between that grid cell and itself. DODs could have been calculated for any number of pairs of grid cells, but was limited to only those pairs corresponding with the empirical genetic data.

We compared the correlations of the pairwise genetic data (*F*_ST_ and *D*_est_) with Euclidean and derived oceanographic distance metrics. Correlations were tested using IBDWS Mantel tests with 10 000 permutations (Jensen *et al*. 2005). To assess sensitivity of the results to the microsatellite loci and sample locations, we also systematically recalculated test statistics leaving out one locus or location at a time from the sample set (i.e. jackknifing, Shao & Tu 1995).

## 3. Results

Tested across all 10 populations, we confirmed that the nine microsatellite loci passed quality control screening for null alleles, linkage disequilibrium and the Ewens–Watterson exact test of neutrality as outlined in White & Toonen (2008). Heterozygosity ranged from 0.18 to 0.91 across loci. Within-population heterozygosity was relatively low and showed little variation across sites at all loci (table 1).

Population genetic structure among all 10 sites was low but significant (global *F*_ST_ = 0.00138, *p* = 0.018; global *D*_est_ = 0.001). Genetic differentiation between pairwise sites ranged from −0.0023 to 0.0068 (*F*_ST_) and from −0.0048 to 0.023 (*D*_est_) (table 1). Six of the 45 pairwise *F*_ST_ values were statistically significant (*p* < 0.05), compared with approximately 2 expected by chance. Results from pairwise *F*_ST_ and *D*_est_ were correlated (*R*^*2*^ = 0.68, *p* = 0.0001; electronic supplementary material, figure S2), and the interpretation of the patterns was unchanged regardless of which measure of population structure was applied. For both *F*_ST_ and *D*_est_, we did not detect a fit with a conventional isolation-by-distance model based on pairwise Euclidean distance (table 2; figure 1). In each of the three simulation sets analysed in program Geneland, the modal value of *K* among 10 replicate simulations indicated that the 709 sampled individuals constitute a single population.

**Table 2.** Summary statistics of linear regression of genetic distance values with Euclidean and derived oceanographic distance (based on multi-generation connectivity probability) between pairwise sites. Correlations with negative genetic distances set to zero are presented in electronic supplementary material, table S2.

		Euclidean distance		oceanographic distance	
sites		*F*_ST_	*D*_est_	*F*_ST_	*D*_est_
all sites	*R*^2^	0.0042	neg slope	0.33	0.24
	*p*	0.32	0.52	0.0055	0.0157
islands	*R*^2^	0.10	0.002	0.47	0.53
	*p*	0.21	0.43	0.034	0.025
mainland	*R*^2^	neg slope	neg slope	0.04	neg slope
	*p*	0.37	0.44	0.32	0.52
cross channel	*R*^2^	0.0041	neg slope	0.44	0.41
	*p*	∼1	∼1	0.007	0.014

**Figure 2.**
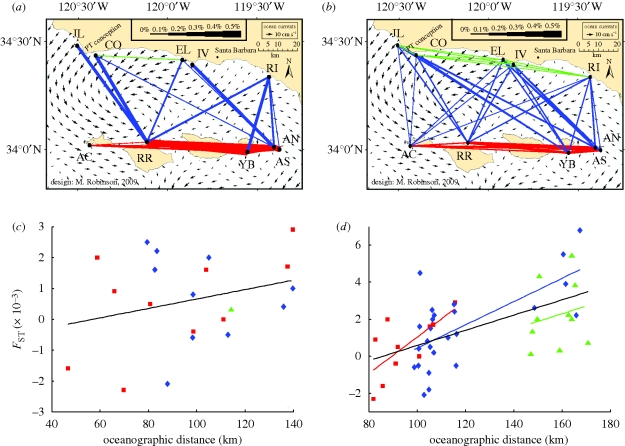
(*a*,*b*) Mean probability of dispersal between genetic sampling sites, overlaying mean surface currents 15 June–15 October 1993–1999 (arrows, size correlates with velocity) in the Santa Barbara Channel. Line thickness correlates with probability. (*a*) Probability of dispersal over a single generation, **M**; (*b*) long-term probability of dispersal over multiple generations, **M**_**ss**_. (*c*,*d*) Genetic differentiation in relation to derived oceanographic distance between sites, based on (*c*) **M** (infinite pairwise distances excluded) and (*d*) **M**_**ss**_ (all pairwise sites included). Red squares, islands; green triangles, mainland; blue diamonds, cross channel. See text and table 2 for regression statistics.

**Figure 3.**
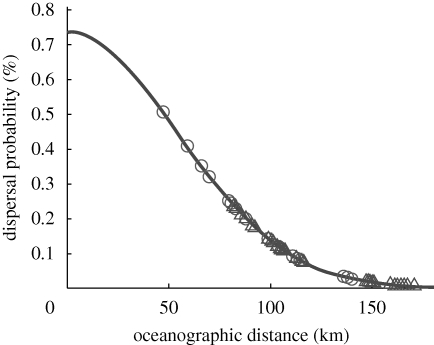
Calculation of derived oceanographic distance in relation to the mean probability of dispersal between pairwise sampling sites (open circles, single generation; open triangles, multi-generation), based on a Gaussian larval dispersal PDF (curved line). **M**, single-generation probabilities (*n* = 21, i.e. excluding 24 zero probabilities). **M**_**ss**_, multi-generation probabilities (*n* = 45, i.e. all pairwise connections). Lower probabilities of dispersal translate into increased derived oceanographic distance pairwise sites.

In the original forward transition matrix (**M**, representing dispersal over a single generation), mean probabilities of dispersal among coastal grid units representing the 10 geo-referenced genetic sampling sites ranged from zero to 0.51 per cent (figure 2*a*). Using Siegel *et al*.'s dispersal kernel, we transformed the probabilities into DODs that ranged from ∞ − 47 km, respectively (figure 3). Infinite distances (i.e. site pairs with zero probability of dispersal, occurring between 24 of the 45 pairs) were excluded, and regression of DOD against pairwise genetic differences was not statistically significant (*F*_ST_: *R*^2^ = 0.08, *p* = ∼1; *D*_est_: *R*^2^ = 0.24, *p* = 0.49; figure 2*c* and electronic supplementary material, figure S3*a*).

The steady-state transition matrix, **M**_**ss**_, showed long-term probability of dispersal between the 10 geo-referenced locations ranging from 0.0051 to 0.23 per cent (figure 2*b*). From these probabilities, we calculated DODs 171–82 km, respectively (table 1 and figure 3; see electronic supplementary material, appendix B, for variance in DODs across the 7 simulation years in the oceanographic model). The Markov chain introduced positive probabilities of dispersal between the 24 locations that were not connected by direct dispersal over a single generation. Because the Markov chain discounts a dispersal process by the number of transitions it contains, these ‘new’ probabilities were smaller than those between sites already connected by direct dispersal (compare line thicknesses in figure 2). Inclusion of the additional probabilities reduced the maximum value in **M**_**ss**_ because the forward matrix is constrained to sum to one for each source. In contrast to Euclidean distance, regression of DOD based on long-term dispersal probability against pairwise genetic difference explained considerable variance in pairwise *F*_ST_ and *D*_est_ values, and was statistically significant (*F*_ST_: *R*^2^ = 0.33, *p* = 0.0055; *D*_est_: *R*^2^ = 0.24, *p* = 0.0157; table 2 and figure 2*d*; electronic supplementary material, figure S3*b*). Jackknifing the data found the observed pattern to be largely independent of any single locus or sampling location (electronic supplementary material, table S1). Although convergence in **M**_**ss**_ took many generations, corresponding with evolutionary time scales, similar regression correlation strengths were obtained when the Markov chain was run for fewer iterations (electronic supplementary material, figure S4). Setting negative *F*_ST_ and *D*_est_ values to zero had a minimal effect on the correlation with DOD, and did not improve the significance of the correlation with Euclidean distance (electronic supplementary material, table S2).

## 4. Discussion

This study demonstrates the value of considering dispersal from the perspective of the organism for estimating population connectivity. Calculation of oceanographic distance in relation to the frequency of dispersal enabled us to construct genetic isolation-by-distance plots that were more effective at explaining population genetic structure than those based on Euclidian distance. Euclidean distance was uncorrelated with the probability of dispersal or DOD (electronic supplementary material, figure S5). Thus, it is not surprising that Euclidean distance was a poor predictor of genetic structure because probabilities of larval exchange, and presumably gene flow, among sites have little to do with the physical distance between them. The decoupling of dispersal probability and DOD from Euclidean distance between sites is probably due to the complex geography and circulation of the region.

We considered the isolation-by-distance regression under three regional groupings of site pairs—those at the islands, mainland and across the Santa Barbara channel (indicated by colours and symbols in the figures). This *post hoc* analysis highlighted variation in how well the consideration of larval dispersal in the oceanographic model explained genetic structure among different groups of sites. For example, model-based probabilities of dispersal were high between several cross-channel pairs linked by a within-channel eddy (figure 2) (Harms & Winant 1998; Dong *et al*. 2009; Mitarai *et al*. 2009), leading to small DODs between these geographically distant sites. Concurrently, probabilities of dispersal were low, and thus DODs high, between Adam's Cove on the westernmost Channel Island (San Miguel Island) and all of the mainland sites (especially Jalama and Coho, near Point Conception). High current velocities common to San Miguel Island and around Point Conception may limit larval settlement there owing to high advection of larvae offshore (Dong & McWilliams 2007). The pattern of DODs across the Santa Barbara channel was reflected in the genetic data; as a result, replacement of Euclidean distance with DOD explained nearly 50 per cent more variance in the genetic differences between cross-channel sites (table 2). Genetic differences between site pairs within the islands were very low and showed a poor fit with the conventional isolation by the Euclidean distance model, a pattern typically interpreted as representing panmixia. However, the isolation-by-distance regression was strong and significant when evaluated in relation to DOD (table 2), indicating that the empirical genetic pattern reflects meaningful differences in connectivity among the island sites. The DOD incorporates the observed ocean flows squeezing between the shallow waters between the Northern Channel Islands (Dong & McWilliams 2007). These flows may limit dispersal in the Channel Islands, and in another island system have also been implicated to increase inter-island genetic differences (Johnson & Black 2006). Interestingly, DOD between sites Anacapa North and Anacapa South, located on opposite sides of long and narrowly shaped Anacapa Island, was low despite their relatively high genetic difference. Derived oceanographic distance was also the most variable across years between these two sites compared with between any other site pairs (electronic supplementary material, appendix B). This outlying result in our study may be an artefact of the coarse spatial resolution of the oceanographic model relative to Anacapa Island's unusual shape and small size. Along the mainland, larval retention was limited in areas with strong advection (thick arrows in figure 2). Consequently, we estimated low probabilities of dispersal, and thus large DODs, among mainland sites. These estimates contributed to the overall regression, but did not explain the observed genetic differences among the mainland sites in particular (table 2). This lack of explanation may reflect challenges in accurately modelling near-shore patterns of ocean flow along the mainland (e.g. Largier 2003).

Our findings based on *F*_ST_ and *D*_est_ were very similar—for both metrics, geographical (Euclidean) distance was a poor explanatory variable compared with DOD. Given the relatively low and equivalent levels of gene diversity observed among the sampling sites (table 1), the data do not fall into the region of parameter space for which a standardized estimate of genetic differentiation is argued to be critical to interpretation (Heller & Siegismund 2009; Jost 2009; Ryman & Leimar 2009). Thus, it is unsurprising that pairwise *F*_ST_ and *D*_est_ values correlated so well (electronic supplementary material, figure S2; Jost 2008) and produced similar results (see electronic supplementary material, figure S4, for an illustration and discussion of subtle differences in results).

The utility in our approach to evaluating genetic isolation by distance was more apparent after we considered multi-generational transition probabilities via the Markov chain—a result corresponding with theory because genetic estimates are presumably based on equilibrium-level connections derived over multiple generations. The difference in results generated using matrices **M** versus **M**_**ss**_ suggests that estimates of direct, single-generation dispersal processes, e.g. via simulation models (Cowen *et al*. 2006; Xue *et al*. 2008), mark–recapture experiments (Jones *et al*. 1999) and genetic (Roques *et al*. 1999; Planes *et al*. 2009) and micro-chemistry assignment tests (White *et al*. 2008), may not by themselves be indicative of the average pattern of connectivity among coastal locations. However, removal of a single outlier, Anacapa North, more than doubled the strength of the correlations (i.e. *R*^2^ values) of *D*_est_ and *F*_ST_ with DOD based on **M**. Consideration of Anacapa North may compromise the strength of the isolation by oceanographic distance regression because the oceanographic model poorly characterizes currents flowing to and from this location. Thus, limitations in the oceanographic model in relation to the experimental design of study sites may have generated results that undervalue the potential for single-generation dispersal probabilities to contribute significantly to the interpretation of population genetic structure. Alternatively, oceanography may simply be less influential on Anacapa Island's genetic structure compared with at other sites. Use of an improved circulation model and consideration of additional environmental factors (e.g. ocean temperature, chemistry or habitat; e.g. Fontaine *et al*. 2007) could resolve the relative influence of these factors on Anacapa Island's genetic structure and the relative importance of single- versus multi-generation dispersal processes in shaping population connectivity.

Our simulation model makes several important simplifying assumptions. Larvae are assumed passive, despite laboratory observations of diel vertical migration in *K. kelletii* larvae (D. C. Zacherl 2009, unpublished data) and demonstrations of the effect of vertical migration on dispersal (Fuchs *et al*. 2004; Marta-Almeida *et al*. 2006; Paris *et al*. 2007). However, the thermocline (where stratification occurs and velocity can change rapidly in speed and direction with depth) in the Southern California Bight is considerably deeper (30–50 m depth) than the lower depth range of many pelagic larvae exhibiting vertical migratory behaviours (e.g. Queiroga & Blanton 2005). Also, above the thermocline, the current profile changes slowly with depth compared with other coastal regions, such as the Caribbean continental shelf (Harms & Winant 1998; Andrade *et al*. 2003; Dong *et al*. 2009). Consequently, in simulations of dispersal patterned after the California Current, incorporation of larval behaviour affecting their vertical movement altered dispersal scales only minimally (Siegel *et al*. 2008). Thus, consideration of vertical migratory behaviour may only have a small influence on our results. Larval production is assumed constant across all coastal sites for the entire spawning season in our model, despite observed variance in *K. kelletii* population density (Zacherl *et al*. 2003). Spatial variability in population abundance, and thus larval output, is expected to change the relative magnitude of larval exchange among sites in our oceanographic model; consideration of such magnitudes may increase estimates of pairwise connectivity involving high-density source populations. We assumed no mortality during dispersal. Positive larval mortality is expected to induce nonlinear reductions across the dispersal probability matrices, especially between distant pairwise locations (Cowen *et al*. 2000; Lefebvre *et al*. 2003; Paris *et al*. 2007). Explicit consideration of larval mortality may improve interpretation of empirical patterns of genetic structure (Graham *et al*. 2008). In transformation step 1, we averaged model results across 7 simulation years, despite temporal variance in DOD values and the strength of the isolation-by-distance correlation across years (electronic supplementary material, appendix B). Additional explanatory power of the population genetic pattern may be gained through explicit consideration of inter-annual variance in DOD values in relation to annual environmental conditions (e.g. El Niño Southern Oscillation index, which correlates positively with annual isolation-by-distance *R*^2^ values; results not shown). In transformation step 4, we represented mean dispersal probability as an average of uni-directional dispersal probabilities. This necessary simplification of the output provided by the oceanographic model is accurate given the isolation-by-distance approach we have taken, but future improvements on this approach might consider asymmetrical uni-directional probabilities of dispersal among sites. Additional explanatory power may be gained using genetics models that estimate directionality in migration, because asymmetry in migration rate can influence genetic structure (Wilkinson-Herbots & Ettridge 2004). Overall, we expect the simplifying assumptions in our analysis to reduce rather than increase the strength of the correlations shown here, and predict that future incorporation of more biologically realistic assumptions will only further increase the utility of this approach.

It is likely that there are many situations where a stepping-stone model is an inadequate framework for describing the gene flow of marine species. A conventional stepping-stone model assumes that diffusion dominates ocean circulation over equilibrium population genetic time scales. However, ocean flow simulations with even simple but real coastlines can generate complex spatial patterns of connectivity whose spatial and temporal average departs considerably from expected Gaussian dispersal kernels based on homogeneous flow conditions (Aiken *et al*. 2007; Pringle & Wares 2007). In the Santa Barbara Channel, heterogeneous flow resulting from persistent nonlinear features such as eddies and island wakes highlights the relevance in using numerical simulations over simple diffusion functions for characterizing larval dispersal (DiGiacomo & Holt 2001; Dong & McWilliams 2007; Mitarai *et al*. 2008). At the core of our analysis is the consideration of these complex circulation dynamics for resolving population genetic structuring that is otherwise interpreted as weak and unintuitive under a conventional stepping-stone model.

This study advances the lower bound of seascape genetics for interpreting fine-scale population structure from seemingly chaotic genetic patchiness characteristic of marine species (Johnson & Black 1984; Muths *et al*. 2009). Despite a 40–60 day pelagic larval duration and an overall *F*_ST_ ≈ *D*_est_ ≈ 0.001, there is significant patterning organized by ocean currents on scales less than 30 km. Furthermore, our results serve as validation for estimating population connectivity using an oceanographic approach, one of the few methods for simulating larval dispersal in marine systems (Baums *et al*. 2006; Galindo *et al*. 2006; Johnson & Black 2006; Levin 2006; Paris *et al*. 2007; Pringle & Wares 2007; Mitarai *et al*. 2008). A similar approach to the interpretation of model outputs on wind dispersal of pollen and seeds may also improve genetic inference for terrestrial plants (Schueler & Schlunzen 2006). Finally, numerous recent studies emphasize that effective marine conservation requires quantifying connectivity patterns among stocks at spatial scales corresponding with fishery management and conservation strategies (Bradbury & Bentzen 2007; Fogarty & Botsford 2007; Weersing & Toonen 2009). Our demonstration of structured gene flow among proximal locations within a small coastal region represents substantial progress towards that goal.
